# Study on cultural representations in mental health and psychosocial support in the Ituri region of the Democratic Republic of Congo

**DOI:** 10.1192/j.eurpsy.2023.381

**Published:** 2023-07-19

**Authors:** E. Dozio

**Affiliations:** Action contre la Faim, Paris, France

## Abstract

**Introduction:**

In the Ituri region of eastern DRC, Djugu territory has been the epicenter of violent clashes in 2020 and significant displacement since 2017. An initial assessment conducted by Action Contre la Faim (ACF) in January 2021 showed a significant level of psychological distress among 85% of respondents. Following this finding, an emergency response was proposed with the aim of contributing to the improvement of the psychological state of men, women, boys and girls affected by displacement and conflict while strengthening their psychological resilience. As part of this response, prior to the intervention, the NGO conducted a study on the representations of mental health and psychosocial needs as well as locally existing support mechanisms.

**Objectives:**

The study aimed to better understand the cultural dimension of the perception of mental health and psychological suffering as well as the use of traditional care or support systems. This will allow for better adaptation of clinical approaches to psychosocial intervention as well as the identification of risks incurred by communities in the context of mental health programming, in a context of inter-community tensions, and the development of mitigation measures, co-constructed with the targeted communities.

**Methods:**

An analysis of strengths, weaknesses, opportunities and threats related to the local perception of mental health issues and pre-existing community support mechanisms was conducted using a mixed methodology. A quantitative approach was used to estimate the prevalence of psychological/psychiatric pathologies present in the intervention area and relate it to known data in the country. This was complemented by qualitative data collection including semi-structured interviews with key informants and focus group discussions with community members (adult men and women) as well as health workers, legal and customary authorities, religious leaders, community leaders, traditional practitioners, humanitarian actors, etc.

**Results:**

12 interviews, 8 focus group discussions and 316 questionnaires confirmed the high rates of distress in the community surveyed.

The cultural representations of mental suffering were better understood (i.e. the origin of suffering and mental illness is exogenous and a spiritual cause is often evoked: witchcraft, curse, divine punishment). Resilience factors (the most resilient would be children and women) and local support mechanisms have been identified, notably in religious leaders.

A very strong group cohesion and solidarity was highlighted.

**Conclusions:**

This study helped to understand the issues related to a mental health care and psychosocial support proposal. This type of study is fundamental to culturally adapting care in humanitarian aid contexts. Concrete details of adaptation will be presented as an example.

**Disclosure of Interest:**

None Declared

